# Women prefer men who use metaphorical language when paying compliments in a romantic context

**DOI:** 10.1038/srep40871

**Published:** 2017-02-09

**Authors:** Zhao Gao, Shan Gao, Lei Xu, Xiaoxiao Zheng, Xiaole Ma, Lizhu Luo, Keith M. Kendrick

**Affiliations:** 1 Key Laboratory for Neuroinformation, School of Life Science and Technology, University of Electronic Science and Technology of China, Chengdu, PR China; 2 School of Foreign Languages, University of Electronic Science and Technology of China, Chengdu, PR China

## Abstract

Language plays an important role in romantic attachment. However, it is unclear whether the structure and topic of language use might influence potential mate choice. We investigated 124 female students’ preference for compliments paid by males incorporating either literal or metaphoric (conventional/novel) language and targeting their appearance or possessions (house) throughout their menstrual cycle. Male faces paired with novel metaphorical compliments were rated as more attractive by women than those paired with literal ones. Compliments targeting appearance increased male attractiveness more than possessions. Interestingly, compliments on appearance using novel metaphors were preferred by women in a relationship during the fertile phase but by single women during the luteal phase. A similar pattern of altered face attraction ratings was subsequently shown by subjects in the absence of the verbal compliments and even though they were unable to recognize the faces. Thus the maintained attraction bias for faces previously associated with figurative language compliments appears to be unconscious. Overall this study provides the first evidence that women find men who typically use novel metaphorical language to compliment appearance more attractive than those using prosaic language or complimenting possessions. The evolutionary significance for such a language use bias in mate selection is discussed.

There has been considerable debate concerning the factors that may have contributed to the evolution of complex human language^1^. Language, as an additional system for social communication and information transmission, is one of the most radical major adaptive changes in human evolutionary terms^2^. Its “digital infinity” in structure that is similar to the genetic system, and “lexical flexibility” in meaning generation are integrated to provide a powerful tool for cultural transmission^3^. However, it remains unclear how such a system emerged and evolved from protolanguage to modern forms that consist of complex syntax and function.

One hypothesis is that the complexity of modern language might have been shaped by a “*mating mind*”^4^ rather than by “*iterated learning*”^3^ or merely by a “*problem-solving mind*”^5^. For humans, according to Miller^4^, language, music, humor, art, etc. are not simply the side-effects of other biological adaptations, but have also evolved through sexual selection pressure to signal a male’s hidden traits of intelligence and creativity. Indeed, studies have consistently demonstrated that intelligence or creativity attributes are preferred by women^6^,^7^,^8^,^9^. Few attempts have been made to support this proposal empirically, although several studies have reported that men who use the most complex and creative language either as poets^6^, or prose writers^9^, have the most female partners. Men’s dating success has also been found to be correlated with their creativity^8^. Furthermore, the linguistic strategy used by men who were given photos of young, attractive women and required to imagine a romantic encounter with them was to display novelty by using less common words^7^.

Undisputedly, linguistic ability is part of cognitive intelligence^10^,^11^, and provides a variety of different ways to express the same meaning, thereby enhancing our creative potential. Figurative language, particularly the use of metaphor, is regarded as a typical linguistic structure that reflects intellectual creativity and wit^12^. Metaphors, which project from one conceptual domain to another, involve higher cognition processes^13^ and greater activation in key brain language and cognitive processing areas such as the inferior frontal gyrus, insula and temporal cortices^14^,^15^,^16^. Nevertheless, not all metaphors require the same magnitude of cognitive load, e.g. those stereotyped metaphors we are constantly exposed to may require similar efforts to produce and comprehend as literal expressions due to their familiarity^13^,^17^. Indeed, use of such conventional metaphors may be less associated with fluid intelligence^12^,^18^ and are distinct from either novel metaphors or literal expressions in terms of difficulty in cognitive processing and ratings of creativity and saliency^19^,^20^. In the context of the current experiment we therefore hypothesized that women would find men using metaphorical language, especially novel metaphors, more attractive.

We generally like people more who praise us by paying verbal compliments^21^. It is well known that compliments play a vital role in development and maintenance of interpersonal relationships, especially in romantic ones. For example, compliment frequency is positively correlated with relationship satisfaction^21^, and complimentary gambits are often used by men to attract the attention of a potential mate^22^,^23^, particularly those targeting their physical appearance^24^. Thus in a courtship context, compliments targeting personal appearance might express a stronger sexual interest than those towards some non-personal attribute such as a person’s possessions.

To date, a woman’s preference for a potential mate has been found to be context-dependent and influenced by multiple factors^25^,^26^. For example, a number of studies have reported that women tend to exhibit a bias towards more masculine faces, voices, body and other physical traits during the fertile phase of their menstrual cycle although more feminine features at other times^27^,^28^,^29^,^30^. Female perception of male attractiveness also varies dependent upon whether the mating context is short- or long-term^31^,^32^, and with their own relationship status^33^. Thus if mate selection is the evolutionary drive for development of creative language, then a woman’s preference for certain linguistic structures and individuals should be influenced most during the fertile phase of her menstrual cycle when “good genes” in a prospective partner become a prerequisite for reproduction. However, findings are still somewhat inconsistent for whether a woman’s preference for intelligence or creativity should be strengthened during her fertile phase or not^34^,^35^. Furthermore, no studies to date have directly investigated the impact of figurativeness in verbal compliments on female preferences in a courtship context.

In the current study we therefore aimed to investigate: 1) Which type of compliment can increase men’s attractiveness in women’s eyes, literal or metaphoric? 2) If women prefer compliments targeting their appearance or possessions? 3) If the preference for figurativeness and topic of compliment varies during the menstrual cycle? Since facial attractiveness is believed to reflect male genetic quality^36^,^37^,^38^, and to create a courtship context between heterosexual strangers^39^,^40^,^41^, in this experiment female participants were required to rate men’s attractiveness based on their facial photos paired with different verbal compliments. The basic design of the experiment was similar to that used to demonstrate the impact of first impressions on social attractiveness by pairing the faces of individuals of average attractiveness with verbal information about their character^42^. To help dissociate effects of the attractiveness of the compliment *per se* from its specific impact on the attractiveness of an individual’s face associated with it, participants rated faces first in conjunction with the verbal compliment and then subsequently in the absence of the latter. On the basis of previous studies we hypothesized that: 1) Men who use metaphoric language to pay compliments, novel metaphors in particular, will be rated as more attractive than those who use literal compliments; 2) Men who compliment a woman’s appearance will be perceived as more attractive than those complimenting her possessions and 3) Any observed attraction bias for the figurativeness or topic of compliments will be strongest during the fertile phase of the menstrual cycle.

## Results

### Independent ratings of the compliments used in the main study

Two independent groups of female subjects rated the compliments used in the main study for either language and appropriateness or perceived characteristics of the male using them. Results are shown in Table S1. As expected both novel and conventional metaphor compliments were rated significantly higher on figurativeness and language attractiveness than literal ones independent of their topic (i.e. appearance or possessions). Similarly, the novel metaphor compliments were rated significantly less familiar than the conventional metaphor or literal ones. Literal compliments targeting appearance were rated slightly more appropriate linguistically than novel metaphors targeting appearance, and novel metaphors scored lower on imageability than conventional metaphor or literal ones. Importantly in the context of the main study there were no significant differences across the six different categories of compliments in terms of their perceived valence (*F* = 0.833, *p* = 0.532, 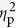 = 0.276) or interpersonal attractiveness (*F* = 1.885, *p* = 0.112, 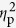 = 0.596). In this case interpersonal attractiveness judgements were made in terms of how subjects rated them as being generally appropriate and attractive in an interpersonal context rather than in terms of a specific personal preference. Thus there were no perceived differences in how positive the different compliments were or their relevance in a general social context. Results from the second group of raters showed a significant relationship between figurativeness and perceived intelligence, with literal compliments targeting either appearance or possessions being rated lower than metaphorical ones. On the other hand there was no significant effect of perceived dominance across the six compliment types (*F* = 0.853, *p* = 0.519, 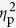 = 0.282). Thus in agreement with previous findings^10^,^11^,^12^, language complexity is particularly associated with perceived intelligence.

### Attractiveness ratings in relation to LAS questionnaire scores

In the main study no group differences were found in questionnaire scores and cycle length between women in the two different relationship status groups (see Table 1).

Two participants did not fully complete the LAS questionnaire and so could not be included in the analysis. Among the six LAS types, female participants predominantly exhibited a primary Storge style (practical or logical love – 45.61%) and a secondary Pragma style (friendship-based love – 68.42%). According to PLS, 86% of female participants in the relationship group were in passionate love and 58.14% of these were wildly and recklessly in love.

A percentage homogeneity test found no significant overall difference between relationship status and LAS (Cramer’s *V* = 0.093 for primary type and 0.132 for secondary type). Even though the single women group had a slightly different profile of attitudes from those of women in a relationship, the proportion of women exhibiting the different love styles in the relationship and singles groups did not differ significantly (see Supplementary Table S2). The proportions of women exhibiting the different LAS types were similar in the different phases of the menstrual cycle.

LAS types were not found to be correlated with the attractiveness ratings for faces paired with the different compliments in the relationship group although for the singles condition there was a correlation between LAS Storge and the attractiveness of conventional compliments targeting possessions (*r* = 0.233, *p* = 0.013).

### Overall effects of compliments on attractiveness ratings

Paired-sample *t* tests revealed that compliments significantly increased attractiveness ratings. In session one, faces paired with verbal compliments (*M* = 4.404, SD = 0.648) were rated significantly more attractive than those presented alone (*M* = 4.258, SD = 0.691), *t*(116) = 2.675, *p* = 0.009, *d* = 0.753. In session two, attractiveness ratings of faces previously paired with compliments were significantly higher than those of unfamiliar control faces (*M* = 4.334, SD = 0.658 vs *M* = 4.144, SD = 0.605; *t*(116) = 7.395, *p* < 0.001, *d* = 1.000). Importantly, there was no significant difference in attractiveness ratings given for the 10 faces which were not paired with compliments between the two sessions (*M*_s1_ = 4.258, SD = 0.691; *M*_s2_ = 4.353, SD = 0.743; *t*(116) = 1.347, *p* = 0.181, *d* = 0.264). Thus familiarity with the faces *per se* is unlikely to have contributed to the difference between ratings given to the familiar faces previously paired with compliments as opposed to unfamiliar faces in session 2.

### Attractiveness ratings in relation to figurativeness and topic of compliments

**Figure 1:**
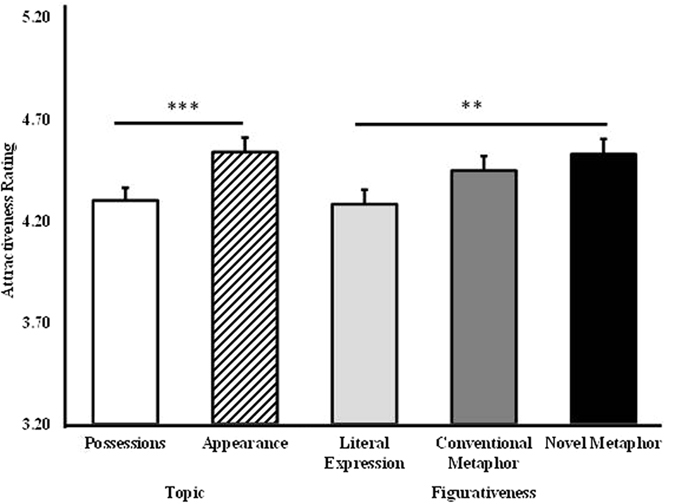
Main effects of topic and figurativeness in session one. The men who either used novel metaphorical compliments or targeted women’s appearance were rated as more attractive than those who used literal expressions or targeted women’s possessions. Bars show means and s.e., **p < 0.01, ***p < 0.001.

ANOVA analysis on attractiveness ratings in session one revealed main effects of figurativeness (*F*_2,224_ = 6.756, *p* = 0.002, 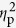 = 0.891) and topic (*F*_1,112_ = 17.029, *p* < 0.001, 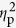 = 0.983) (see Fig. 1). The faces paired with novel metaphorical compliments relative to literal ones (*p* = 0.005) and compliments on a woman’s appearance relative to her possessions (*p* < 0.001) were rated significantly higher. There were however no statistically significant differences in attractiveness ratings between conventional and novel metaphors (*p* = 0.392). Experience of being in love *per se* did not confound the result since in the singles group there was no significant difference between the ratings of women who had previously been in a relationship, but were now single (N = 25), and the women who had never had a relationship (N = 40), *t*(65) = 1.091, *p* = 0.279, *d* = 0.184).

**Figure 2:**
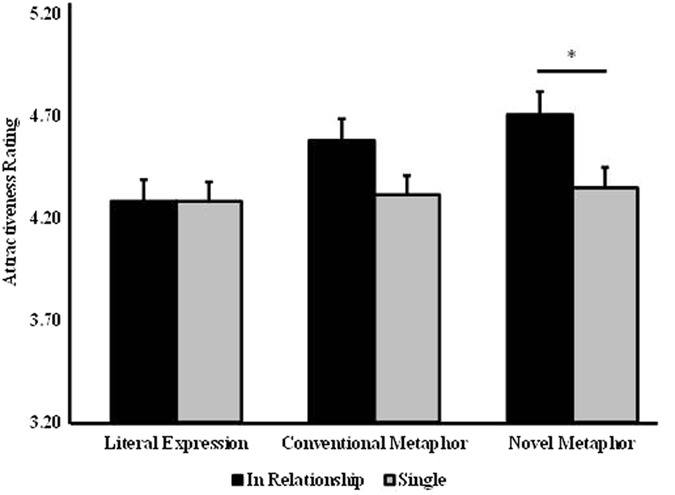
Two-way interaction between romantic relationship status and figurativeness of compliments in session one. The men who used novel metaphorical compliments were rated significantly more attractive by women in a relationship than by single women. Bars show means and s.e., *p < 0.05.

**Figure 3:**
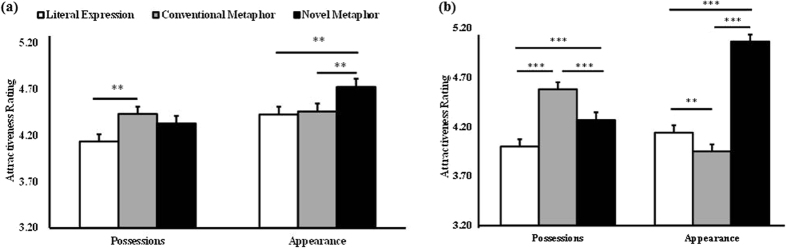
Two-way interaction between the topic and figurativeness of compliments. (**a**) In session one, the men who used conventional metaphors were rated as more attractive than those who used literal expressions when targeting women’s possessions, whereas when targeting women’s appearance, the men who used novel metaphorical compliments were rated as most attractive. (**b**) In session two, the men who had previously used conventional metaphorical compliments were rated most attractive when targeting women’s possessions, whereas the men who had previously used novel metaphorical compliments were rated as most attractive when targeting women’s appearance. Bars show means and s.e., **p < 0.01, ***p < 0.001.

Significant two-way interactions were found for: figurativeness x relationship (*F*_2,224_ = 3.755, *p* = 0.025, 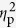 = 0.682), and figurativeness x topic (*F*_2,224_ = 6.594, *p* = 0.002, 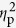 = 0.908). *Post hoc* comparisons showed that women in a relationship had a greater overall preference for figurativeness relative to the single women, although this only achieved significance for the novel metaphorical compliments (*p* = 0.018; see Fig. 2). For compliments targeting possessions conventional metaphorical compliments were preferred to literal ones (*p* = 0.002) whereas for those targeting appearance the novel metaphorical compliments were preferred to both literal (*p* = 0.002) and conventional metaphor ones (*p* = 0.005) (see Fig. 3a).

**Figure 4:**
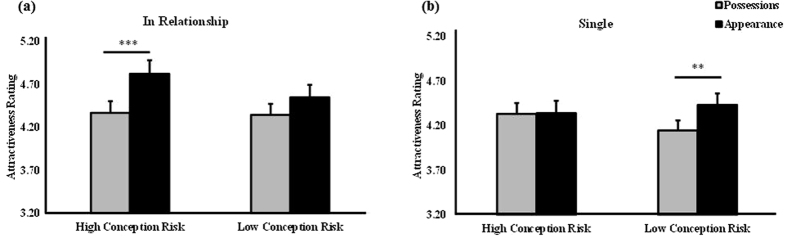
Three-way interaction between romantic relationship, menstrual cycle and compliment topic in session one. (**a**) The men who complimented women’s appearance were rated as more attractive than those who complimented their possessions by women in a relationship during the high conception risk phase of their cycle. (**b**) The men who complimented women’s appearance were rated as more attractive than those who complimented their possessions by single women during the low conception risk phase of their cycle. Bars show means and s.e., **p < 0.01, ***p < 0.001.

There was also significant topic x relationship x conception risk interaction (*F*_1,112_ = 5.274, *p* = 0.024, 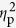 = 0.624). *Post-hoc* multiple comparisons showed that the men who complimented women’s appearance were rated more attractive than those who complimented their possessions by women in a relationship during the high conception risk phase but by single women in the low conception risk phase (Relationship + High conception risk: *M*_appearance_ = 4.826, SE = 0.156, *M*_possessions_ = 4.371, SE = 0.136; Single + Low conception risk: *M*_appearance_ = 4.435, SE = 0.129, *M*_possessions_ = 4.150, SE = 0.112). In other words, women’s relationship status and conception risk influenced their preference for the topic of men’s compliments (see Fig. 4).

Recognition accuracy calculated at http://memory.psych.mun.ca/models/recognition/ (d’ = 0.796 < 4.65 sensitivity threshold, C = 0.844) indicated a conservative bias that female participants were less likely to identify faces as familiar. Indeed, they failed to exhibit recognition memory for the stimulus faces they had viewed and rated for attractiveness in session one (hit rate is 32%).

The attraction ratings in session two were also analyzed in order to see if the different types of compliments paired with individual faces influenced subsequent attractiveness of these faces when they were presented alone. Results showed that the attraction ratings for the faces originally paired with compliments on appearance in session one were also significantly higher in session two than those on possessions (*M*_appearance_ = 4.384, SE = 0.063; *M*_possessions_ = 4.281, SE = 0.065; *F*_1,112_ = 10.403, *p* = 0.002, 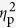 = 0.892) and also for those using metaphors as opposed to literal language (*M*_novel metaphor_ = 4.665, SE = 0.068; *M*_conventional metaphor_ = 4.264, SE = 0.062; *M*_literal_ = 4.069, SE = 0.069; *F*_2,224_ = 114.011, *p* < 0.001, 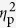 = 1.000). There was a significant topic x figurativeness interaction (*F*_2,224_ = 142.347, *p* < 0.001, 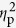 = 1.000) due to faces originally paired with novel metaphors complimenting appearance and those paired with conventional metaphors complimenting possessions being rated as more attractive (*M*_novel metaphor appearance_ >*M*_conventional metaphor appearance_ >*M*_literal appearance_, *p*s < 0.001; *M*_conventional metaphor possessions_ >*M*_novel metaphor possessions_ >*M*_literal possessions_, *p*s < 0.002. See Fig. 3b). The results of paired-sample *t* tests revealed that the difference in attractiveness ratings between novel metaphors and the other two forms of figurativeness targeting appearance in session two is significantly higher than in session one (Rating difference between novel and conventional metaphor for appearance in session two = 1.128 and in session one = 0.271, *t*(116) = 8.822, *p* < 0.001; Rating difference between novel metaphor and literal for appearance in session two = 0.928 and in session one = 0.288, *t*(116) = 6.039, *p* < 0.001). However, no effects of cycle phase were found in the second session.

Thus, overall findings in the second session of the study were consistent with the attraction bias generated in the first session being maintained, albeit without participants consciously being able to remember the faces. Nevertheless, the pattern of effects did differ somewhat between the two sessions suggesting that the presence of the verbal compliments *per se* in session 1, but not in session 2, may have had some influence on attractiveness ratings given by the two different relationship groups.

## Discussion

This preliminary study aimed to explore whether language usage could influence mate selection, which could support the possibility that language evolution has been driven by sexual selection. This is the first study to directly examine a potential evolutionary bias for the usage of different types of linguistic forms. In our experiment a typical mate selection scenario was created for female participants using male faces of average attractiveness^22^,^23^,^40^,^41^,^43^ which were additionally paired with verbal compliments varying in terms of figurativeness and topic. Using this approach differences in attractiveness rating scores should be primarily driven by linguistic variance and topic rather than by the faces *per se*. The findings confirm our hypotheses that in a courtship situation where compliments serve as a sexual display of mate quality, women show a preference for metaphors, particularly novel ones, in verbal structure. In agreement with previous studies^6^,^7^,^8^,^9^ we found that the use of metaphorical as opposed to prosaic language by men in compliments is perceived by women as indicative of creativity and intelligence. The preference observed for metaphorical compliments targeting a woman’s appearance compared to possessions may be indicative that this generates greater sexual attraction towards a potential mate. Furthermore, this impact of language usage in compliments on attractiveness judgments does not vary with women’s love style attitudes. Interestingly, while participants were subsequently unable to remember the faces paired with different types of compliments better than chance, when the faces were presented alone, attraction ratings for those previously paired with metaphorical compliments continued to be rated as the most attractive. This indicates that the previous association with such metaphorical compliments resulted in an unconscious attraction bias towards these individuals.

Overall, these results support our first hypothesis that men who use metaphorical compliments, particularly novel ones, are perceived as more attractive by women than men who produce more prosaic literal compliments. Previous studies have revealed that compared with other mate qualities women, in contrast with men, prefer creativity and intelligence rather than physical attractiveness^44^,^45^,^46^,^47^, and for the compliments used in our current study women did indeed perceive those which were figurative as indicating higher intelligence in a man than literal expression compliments. Interestingly use of figurative as opposed to literal language in compliments was not significantly linked to perceived dominance which is also known to influence male attractiveness. Thus the type of language use during interactions with a prospective mate may provide a key indicator of a male’s intelligence and creativity although not dominance. We tend to form very rapid impressions about a person’s attractiveness in social contexts^42^ and thus for women, cues from language usage during initial encounters may provide a rapid first assessment of a potential mate’s intellectual and creative abilities. The current study deliberately paired verbal compliments with the faces of men of average attractiveness in order to optimize the chances of showing an impact of language usage on perceived attractiveness. It therefore remains to be seen whether metaphorical language usage would also increase the attractiveness of individuals with either low or high facial attractiveness.

We tried in the current study to control for possible confounding contributions of non-specific factors unrelated to language on altered attractiveness ratings. The face stimuli used were deliberately standardized and of average attractiveness in order to render them as homogeneous as possible and pairing of faces with specific compliment sentences was entirely random across subjects. We found no evidence for increased attractiveness ratings of face pictures *per se* when they were presented for a second time, and therefore more familiar. While a previous study has reported that familiarization with unfamiliar faces through repeated exposure can lead to increased attraction ratings^48^, this used a smaller number of faces with more emotive, smiling faces and also a longer exposure time with more frequent presentations than in our current study. It could perhaps be argued that the differential novelty of the sentences paired with faces in the first session might have in some way resulted in the faces associated with them exhibiting increased familiarity/depth of encoding and therefore continuing to be rated as more attractive. However, while the faces originally paired with novel metaphors targeting appearance did indeed receive the highest attractiveness ratings in the second session, those paired with conventional metaphors were significantly less than for literal expression compliments, despite being more novel. Similarly, for compliments targeting possessions, attractiveness ratings for faces associated with novel metaphors became significantly less than those for the more familiar conventional ones in session two (see Fig. 4b). Finally, we also found no evidence for faces associated with either novel or conventional figurative language compliments being better remembered than those paired with literal expressions. We therefore consider that at this stage the most parsimonious explanation for our findings is in terms of the impact of the language content of the verbal compliments on attractiveness ratings of the face pictures rather than as a result of factors unrelated to language such as differential familiarity/depth of encoding. However, further experiments will be needed to completely disentangle all possible contributing factors.

Our second hypothesis that men who complimented women’s appearance as opposed to her possessions would be perceived as more attractive is also supported since in both experimental sessions attractiveness ratings were higher for the faces of individuals paired with compliments targeting appearance. Since ratings of the general social relevance and valence of the compliments targeting possessions as opposed to appearance did not differ significantly it is unlikely that differences in attraction ratings in the main experiment can simply be explained in terms of greater social appropriateness or positivity. The importance of compliment topic can however be both gender- and context- dependent^49^,^50^,^51^. For example, in a same-sex unstructured context women preferred compliments on their appearance whereas men preferred them on their sporting performance^50^. However, our study corroborates previous findings that personal appearance is the most preferred target for compliments in opposite-sex interactions among Chinese people^52^,^53^. Since compliments between the sexes on appearance can easily develop into “flirtation”^48^, they tend to indicate direct sexual intent and are thus likely to provoke reciprocal compliments by recipients. Moreover, the finding that the impact of novel metaphors is weaker when the compliment topic is less sexually directed might indicate that “sexual selection shapes language’s content more than its form”^4^. Thus we found in session one that compliments on appearance were consistently more effective in increasing attractiveness ratings compared to those on possessions, regardless of the type of language used, although in the second session where only faces were viewed this was only the case for individuals previously associated with novel metaphor usage. Possibly novel metaphors targeting appearance might be viewed as particularly “flirtatious” relative to conventional ones, although this would require verification.

Our third hypothesis that the observed attraction bias for the figurativeness or topic of compliments would be greater during the fertile phase of the menstrual cycle was only partially supported. High conception risk greatly increased men’s attractiveness in the eyes of women in a relationship if appearance was praised, although there was a tendency for them to find men who complimented their appearance more attractive across the whole cycle. For the single women on the other hand this preference was exhibited only by those with a low conception risk. The finding for women in a relationship possibly indicates their extra-pair interest when fertile, i.e. for the sake of promoting good genes in their offspring, and thus they show greater attraction for males other than their partners with qualities indicative of having good genes when near ovulation^54^,^55^. However, the single women with high conception risk showed equivalent preference for the men who paid compliments regardless of topic. This suggests that when the single women were in their fertile phase they were not particularly sensitive to the type of compliment given. Possibly this may reflect a tendency to view men who pay any compliments to women as being more likely to show sexual interest in them. On the other hand during the luteal phase when there is no likelihood of conception, and sexual interest is reduced, then they are more selective and compliments targeting appearance become more salient and attractive to them.

Although relationship status influenced the impact of menstrual cycle phase on the attractiveness of compliments, our overall findings showed that attractiveness judgments exhibited a very similar preference pattern for metaphors in both the singles and relationship groups. The only significant difference between the groups was that women in a relationship gave slightly higher ratings than single women did to novel metaphorical compliments in session one, although not in session two. This might suggest that women in a relationship paid more attention to the verbal compliments presented in session one than single women did. This conclusion is supported by a previous study showing that women in a relationship showed a greater attentional bias to flirtatious courtship distractors than single women did^56^. Single women generally exhibited less sensitivity towards compliment topic and form than the women in relationship, particularly in terms of the preference for novel metaphors.

The fact that Pragma (practical) and Storge (friendship-based) love style attitudes constituted 59.65% and 45.61% of the participants in our study may reflect both the participants’ sex^57^,^58^ and Chinese culture^59^. This finding is consistent with the report that Chinese women, relative to their western counterparts, view love as a companionship and place marriage over love^59^,^60^,^61^. Since few correlations were found between the language types of compliments and love attitudes, the preference for use of figurative language in courtship may extend across all love styles and not be modulated by whether individuals exhibit a more pragmatic or romantic love style. However, this would need to be investigated in more detail in a future larger study.

Importantly, the findings in the current study support the view that an attraction bias towards individuals using figurative language for paying compliments is essentially an unconscious one. Thus the female participants in the study provided no overt evidence of improved recognition memory for such individuals or the actual compliments they used, but nevertheless exhibited an attraction bias towards them when presented with their face pictures alone. This is consistent with other research demonstrating the power of “first impressions” where altered behavioral preferences for face pictures of individuals paired with verbal statements about their attributes also failed to result in their subsequently becoming more memorable^42^. Thus both language use and information about an individual’s personal attributes can profoundly alter their perceived attraction, but without someone necessarily being consciously aware of their bias towards them. This is similar in many ways to the influence of “sexual imprinting” where individuals exhibit a learned, but unconscious, attraction bias either towards (positive), or against (negative) specific visual or odor characteristics associated with an opposite sex parent or caregiver^62^,^63^.

There are some limitations for this preliminary study. Firstly, we did not carry out hormone assays to confirm the accuracy of estimations based purely on menstruation dates and self-reported cycle length. Additionally, a within-subject design where women were tested twice at different stages of their cycle may have been more effective in demonstrating menstrual cycle effects. Secondly, in terms of demonstrating effects of relationship status we combined data from single individuals who had both no experience of previous love relationships and those who had had the experience in the past. While we found no significant difference between these two types of individuals in terms of attraction ratings we cannot rule out the possibility that prior love experience might have had some effects. Finally, the current study also only included possessions and facial appearance as compliment topics and further studies need to investigate a wider range of targets including both visual and personality attributes as well as cultural influence^64^ and flirtatiousness of language usage^48^.

In summary the current study has provided preliminary experimental support for the possibility that language evolution in terms of figurativeness may have been influenced by its role in signaling reproductive fitness in the context of mate selection. Future more extensive studies are required to explore this intriguing possibility more fully.

## Methods

### Participants

Participants were 124 heterosexual female college students (mean age = 20.69 years, SD = 2.07) were recruited from different majors at the University of Electronic Science and Technology of China. All participants were experiencing regular, natural menstrual cycles with none taking oral contraceptives. A total of 8 participants were excluded after being identified as statistical outliers by SPSS in terms of either very short response times (mean < 1 s; N = 2) or giving very low attraction ratings indicative of considering the stimuli to be very unattractive (N = 6). Thus finally the data from a total of 116 female participants were analyzed (*M*_age_ = 20.64, SD = 2.07). In this experiment, a continuous average conception probability employing both forward and backward calculation methods was used to classify conception risk: “high conception risk” (with the conception probability ≥0.07) vs. “low conception risk” (with the conception probability <0.07)^65^,^66^. According to relationship status and menstrual cycle information, female participants were stratified into relationship (in-relationship duration of ≥3 months) or single groups (individuals with no previous relationship experience or who had had a relationship that had broken up at least 3 months previously – mean = 11.42 months, SD = 13.18) with high or low conception risk.

Participants first completed Chinese versions of the following questionnaires: EQ^67^, Beck Depression Inventory (BDI^68^), Marlowe-Crowne Social Desirability Scale-Chinese (MCSDS-C^69^), Social Esteem Scale (SES^70^), Love Attitude Scale (LAS^71^). The LAS has high validity among Chinese people with subscale Cronbach alpha values between 0.706 and 0.818^57^,^58^, Passionate Love Scale (PLS^72^). Participants also provided demographic information including age, years of education, romantic relationship status and menstrual cycle (see Table 1).

The present study had full ethical approval from the local ethics committee at the University of Electronic Science and Technology of China and in accordance with relevant guidelines and regulations. Every participant signed informed consent forms before the experiment, and was paid 40 CNY and briefly interviewed at the end of experiment.

## Experimental Stimuli

### Verbal stimuli

A total of 163 verbal compliments were generated by an independent sample (36 males and 9 females), targeting five parts of either a woman’s face (appearance) or her house (possessions): eyes or windows, lips or door, hair or roof, face or house, smile or garden. Each sentence was matched across all conditions for length (average length = 9.6 characters, range: 9–10 characters) and word frequency (mean = 161831.43, SD = 47021.91) according to CCL Corpus (version: contemporary Chinese) provided online by Center for Chinese Linguistics PKU at http://ccl.pku.edu.cn:8080/ccl_corpus. All the sentences were categorized into three kinds of figurativeness (novel metaphor, conventional metaphor, or literal expression) and two topics (appearance or possessions), thus resulting in 6 compliment conditions: novel metaphor on appearance (nma), conventional metaphor on appearance (cma), literal expression on appearance (lea), novel metaphor on possessions (nmp), conventional metaphor on possessions (cmp) and literal expression on possessions (lep). For example, *Your eyes are morning dew* or *Your smile is a naughty goblin* is from the category of conventional metaphors on appearance; *Your roof is a lover’s shoulder* or *Your garden is the sea of flowers* belongs to the category of novel metaphors on possessions; *Your lips are so sexy* or *Your door is very strong* are literal expressions. All sentences were rated using 7-point Likert scales by two independent samples. The first sample (38 female undergraduates, mean age = 19.58 years, SD = 1.55) rated the content and appropriateness of the sentences in terms of figurativeness, familiarity, appropriateness, valence, imageability, language attractiveness, and interpersonal attractiveness (how attractive and appropriate they are rated generally in an interpersonal context)^16^,^73^. The second sample (41 female undergraduates, mean age = 20.71 years, SD = 1.71; 19 high and 22 low-conception risk) rated the sentences in terms of the characteristics of the male using them i.e. how dominant or intelligent.

Verbal compliments rated higher than 4 in figurativeness but lower than 4 in familiarity were categorized into the group of novel metaphors whereas conventional metaphors were those rated higher than 4 in both figurativeness and familiarity. Literal expressions were those for which figurativeness was rated lower than 3. One-way analysis of variance (ANOVA) and *post hoc* Bonferroni tests were used to examine group differences statistically (see Supplementary Table S1). There was no difference in the interpersonal attractiveness of the different compliments in the experiment itself which focused primarily on the attractiveness of verbal compliments in the context of social communication rather than in terms of linguistic attractiveness *per se*. Consequently, the sixty verbal compliments used were balanced in accordance with the seven different criteria and assigned randomly to 60 different male faces. As a control, a further 10 faces were not paired with verbal stimuli.

### Face stimuli

A total of 170 color photographs of male students taken on the day of enrollment were obtained, after informed consent from all subjects at the Registration Office for National College English Tests. The pictures were pre-processed with Photoshop CS6.0 (Adobe System Inc.) to standardize them by removing hair, covering visible clothing and changing the background color to black. The color contrast, brightness, pixels number and size were unified as well. Overall the objective was to produce average attractiveness faces which were uniform in appearance and therefore as homogeneous as possible in term of visual cues which might additionally influence attractiveness ratings.

Emotional valence and facial attractiveness were pre-rated from 1 (*strongly unattractive*) to 9 (*strongly attractive*) by 15 females who did not participate in the experiment (mean age = 19.51 years, SD = 0.88). One hundred and forty face pictures with 3.5–4.5 attractiveness ratings were selected as facial stimuli, and randomly categorized into two groups: experiment faces (*M*_attractiveness_ = 4.151, SD = 0.297; *M*_valence_ = 5.064, SD = 0.240) and control faces (*M*_attractiveness_ = 4.198, SD = 0.287; *M*_valence_ = 5.017, SD = 0.256). No group differences were found for either facial attractiveness, *t* (138) = −0.945, *p* = 0.346 or valence, *t* (138) = 1.128, *p* = 0.261. Next, the 70 experiment faces were each randomly paired either with one of the verbal compliments (i.e. 6 categories with 10 faces in each) or nothing (10 faces).

### Experimental procedures

**Figure 5:**
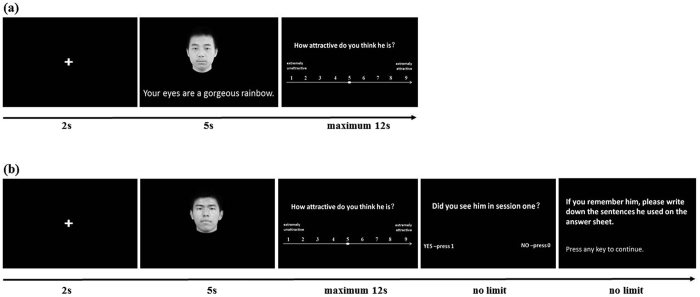
Examples of trials in the two test sessions. (**a**) In session one, a male face randomly paired with a verbal compliment is presented for 5 seconds and his attractiveness is rated afterwards. (**b**) In session two, the faces from session one, or novel faces, are presented alone with no compliments and subjects first rate their attractiveness and then whether they saw the face previously in session 1. The verbal compliments were presented in simplified Chinese but have been translated into English for convenience.

On the day of experiment, the female participants first completed all the questionnaires and then sat at a comfortable distance to a computer display on which stimuli were presented with Eprime 2.0. They were told that each of these male participants had been asked to write down a complimentary sentence after imagining a first visit to a future girlfriend’s house. The experiment consisted of two sessions with one immediately following the other. Face stimuli in the first session were paired with the 60 different compliments or displayed alone (n = 10) in a random sequence for each individual participant (i.e. full randomization). In the second session all the faces shown in session one and an additional 70 novel faces were also shown in a different random sequence for each participant. In both sessions random sequences were generated using Eprime. The first session lasted for about 10 minutes with a maximum of 5 s allowed to make attractiveness ratings from 1 (*very unattractive*) to 9 (*very attractive*) for each of the 70 male face photographs which were either paired with a verbal compliment (n = 60) or presented alone (n = 10). In the second session, the same 70 faces were presented again but without verbal compliments and randomly intermixed with an additional 70 novel control faces. After each picture participants were required to first rate the attractiveness of the individual (1–9 – up to 5 s to make a response) and next to judge whether they were familiar or not (i.e. had the participants seen them during session 1 or not). Finally, participants were asked to write down if they remembered any aspect of the verbal compliment associated with familiar faces. There was no time limit for the familiarity judgment and compliment content components of the task. See Fig. 5 for an example of trials in the two sessions.

### Data analysis

All categories of ratings are normally distributed or log transformed to normal distribution according to Kolmogorov-Smirnov test (*p*s > 0.262). Pearson correlation was used to explore potential associations between attraction ratings and questionnaire scores. For the 60 experiment faces paired with compliments in session one a repeated ANOVA in SPSS 21.0 was used to analyze the factorial design of 2 topics (appearance vs. possessions) × 3 figurativeness (novel metaphor vs. conventional metaphor vs. literal expression) × 2 relationship status (in relation vs. single) × 2 conception risk (high vs. low). In session two when only faces were presented, the faces shown in session 1 (familiar) were stratified into the same 6 groups according to the compliment topic and figurativeness they had originally been paired with. Attractiveness rating data in the two sessions were analyzed using paired t-tests to: 1) compare ratings of the experiment faces shown without compliments in the two sessions to assess potential familiarity effects and 2) compare the ratings in session one between faces paired with compliments and those shown alone and compare the ratings in session 2 between faces originally paired with compliments and novel control faces in order to assess the impact of the verbal compliment on ratings, and 3) compare the difference between novel metaphors and the other two forms of figurativeness targeting appearance in session one with the counterparts in session two. Multiple comparisons were all Bonferroni corrected with *p* < 0.05 considered to be significant.

## Additional Information

**How to cite this article:** Gao, Z. *et al*. Women prefer men who use metaphorical language when paying compliments in a romantic context. *Sci. Rep.* **7**, 40871; doi: 10.1038/srep40871 (2017).

**Publisher's note:** Springer Nature remains neutral with regard to jurisdictional claims in published maps and institutional affiliations.

## Supplementary Material

Supplementary Materials

## Figures and Tables

**Table 1:** Demographic information, cycle stage and questionnaire scores for female participants in the two groups.

	Relationship Group^a^ (N = 51, 24 fertile phase)		Single Group (N = 65, 30 fertile phase)		*t*	*p*
	Mean	SD	Mean	SD		
Age (years)	20.59	2.13	20.68	2.05	0.23	0.82
Cycle length	30.52	3.08	31.13	5.16	0.73	0.47
Education (years)	15.06	1.90	15.14	1.89	0.23	0.82
EQ	40.49	11.58	41.28	9.30	0.41	0.69
BDI	8.31	6.29	8.15	6.55	0.13	0.90
MCSDSS	27.90	3.68	26.91	3.45	1.50	0.14
SES	31.37	4.40	31.22	4.62	0.19	0.85
Relationship length (months)	20.78	19.12				
Intensity of love (PLS score)	102.76	16.80				

^a^Minimum relationship duration = 3 months.
