# Supplementary material for: Women prefer men who use metaphorical language when paying compliments in a romantic context
Source: Sci Rep. 2017 Feb 9;7:40871. doi: 10.1038/srep40871 (PMC5299994; doi:10.1038/srep40871)
Supplement: Supplementary Materials [file srep40871-s1.doc]

Women prefer men who use metaphorical language when paying compliments in a romantic context

Zhao Gao^1,2^, Shan Gao^1,2^, Lei Xu^1^, Xiaoxiao Zheng^1^, Xiaole Ma^1^, Lizhu Luo^1^, Keith M. Kendrick^1*^

1. Key Laboratory for Neuroinformation, School of Life Science and Technology, University of Electronic Science and Technology of China, Chengdu, PR China
2. School of Linguistics and Literature, University of Electronic Science and Technology of China, Chengdu, PR China

Supplementary Table S1

| **Table S1** ▏**ANOVA analysis of ratings for the three types of compliment stimuli** | | | | | | | | | | | | | | | | | | |  |
| --- | --- | --- | --- | --- | --- | --- | --- | --- | --- | --- | --- | --- | --- | --- | --- | --- | --- | --- | --- |
| **Criteria Types** | | | **N** | | | **Mean** | | | **Std. Deviation** | | | ***F* & *p*** | | | | **Significant^b^ (Bonferroni)** | | |  |
| Figurativeness | | 1-nma**^a^** | 10 | | | 5.47 | | | 0.43 | | | *F* = 163.307 *p* < 0.001  *η*2 p= 1.000 | | | | 1/2/4/5 > 3/6 | | |  |
|  |  | 2-cma | 10 | | | 5.36 | | | 0.26 | | |  |  |  |  |  |  |  |  |
|  |  | 3-lea | 10 | | | 2.30 | | | 0.42 | | |  |  |  |  |  |  |  |  |
|  |  | 4-nmp | 10 | | | 5.16 | | | 0.46 | | |  |  |  |  |  |  |  |  |
|  |  | 5-cmp | 10 | | | 5.31 | | | 0.45 | | |  |  |  |  |  |  |  |  |
|  |  | 6-lep | 10 | | | 2.25 | | | 0.28 | | |  |  |  |  |  |  |  |  |
|  | | | | | | | | | | | | | | | | | | |  |
| Familiarity | | 1-nma | 10 | | | 3.35 | | | 0.53 | | | *F* = 40.781 *p* < 0.001 *η*2 p = 1.000 | | | | 2/3/5/6 > 1/4 3 > 2 | | |  |
|  |  | 2-cma | 10 | | | 4.67 | | | 0.13 | | |  |  |  |  |  |  |  |  |
|  |  | 3-lea | 10 | | | 5.29 | | | 0.28 | | |  |  |  |  |  |  |  |  |
|  |  | 4-nmp | 10 | | | 3.24 | | | 0.57 | | |  |  |  |  |  |  |  |  |
|  |  | 5-cmp | 10 | | | 4.74 | | | 0.42 | | |  |  |  |  |  |  |  |  |
|  |  | 6-lep | 10 | | | 4.81 | | | 0.42 | | |  |  |  |  |  |  |  |  |
|  | | | | | | | | | | | | | | | | | | |  |
| Appropriateness | | 1-nma | 10 | | | 4.44 | | | 0.36 | | | *F* = 2.938 *p* = 0.020 *η*2 p = 0.815 | | | | 3 > 4 | | |  |
|  |  | 2-cma | 10 | | | 4.57 | | | 0.66 | | |  |  |  |  |  |  |  |  |
|  |  | 3-lea | 10 | | | 5.08 | | | 0.36 | | |  |  |  |  |  |  |  |  |
|  |  | 4-nmp | 10 | | | 4.23 | | | 0.83 | | |  |  |  |  |  |  |  |  |
|  |  | 5-cmp | 10 | | | 4.94 | | | 0.74 | | |  |  |  |  |  |  |  |  |
|  |  | 6-lep | 10 | | | 4.81 | | | 0.39 | | |  |  |  |  |  |  |  |  |
|  | | | | | | | | | | | | | | | | | | |  |
| Valence | | 1-nma | 10 | | | 5.10 | | | 0.73 | | | *F* = 0.833 *p* = 0.532 *η*2 p = 0.276 | | | |  | | |  |
|  |  | 2-cma | 10 | | | 5.29 | | | 0.57 | | |  |  |  |  |  |  |  |  |
|  |  | 3-lea | 10 | | | 5.38 | | | 0.41 | | |  |  |  |  |  |  |  |  |
|  |  | 4-nmp | 10 | | | 5.08 | | | 0.51 | | |  |  |  |  |  |  |  |  |
|  |  | 5-cmp | 10 | | | 5.41 | | | 0.56 | | |  |  |  |  |  |  |  |  |
|  |  | 6-lep | 10 | | | 5.03 | | | 0.62 | | |  |  |  |  |  |  |  |  |
|  | | | | | | | | | | | | | | | | | | |  |
| Imageability | | 1-nma | 10 | | | 4.08 | | | 0.60 | | | *F* = 7.702 *p* < 0.001 *η*2 p = 0.999 | | | | 1 < 2/3/5 3 > 4/6 | | |  |
|  |  | 2-cma | 10 | | | 4.97 | | | 0.53 | | |  |  |  |  |  |  |  |  |
|  |  | 3-lea | 10 | | | 5.42 | | | 0.41 | | |  |  |  |  |  |  |  |  |
|  |  | 4-nmp | 10 | | | 4.49 | | | 0.53 | | |  |  |  |  |  |  |  |  |
|  |  | 5-cmp | 10 | | | 5.22 | | | 0.64 | | |  |  |  |  |  |  |  |  |
|  |  | 6-lep | 10 | | | 4.61 | | | 0.67 | | |  |  |  |  |  |  |  |  |
|  | | | | | | | | | | | | | | | | | | |  |
| Language Attractiveness | | 1-nma | 10 | | | 5.07 | | | 0.60 | | | *F* = 12.267 *p* < 0.001 *η*2 p = 1.000 | | | | 6 < 1/2/3/4/5 3 < 2 | | |  |
|  |  | 2-cma | 10 | | | 5.10 | | | 0.46 | | |  |  |  |  |  |  |  |  |
|  |  | 3-lea | 10 | | | 4.37 | | | 0.46 | | |  |  |  |  |  |  |  |  |
|  |  | 4-nmp | 10 | | | 4.56 | | | 0.51 | | |  |  |  |  |  |  |  |  |
|  |  | 5-cmp | 10 | | | 4.69 | | | 0.63 | | |  |  |  |  |  |  |  |  |
|  |  | 6-lep | 10 | | | 3.50 | | | 0.49 | | |  |  |  |  |  |  |  |  |
|  | | | | | | | | | | | | | | | | | | |  |
| Interpersonal Attractiveness | | 1-nma | 10 | | | 4.70 | | | 0.62 | | | *F* = 1.885 *p* = 0.112 *η*2 p = 0.596 | | | |  | | |  |
|  |  | 2-cma | 10 | | | 5.09 | | | 0.43 | | |  |  |  |  |  |  |  |  |
|  |  | 3-lea | 10 | | | 4.76 | | | 0.67 | | |  |  |  |  |  |  |  |  |
|  |  | 4-nmp | 10 | | | 4.49 | | | 0.79 | | |  |  |  |  |  |  |  |  |
|  |  | 5-cmp | 10 | | | 4.86 | | | 0.70 | | |  |  |  |  |  |  |  |  |
|  |  | 6-lep | 10 | | | 4.29 | | | 0.59 | | |  |  |  |  |  |  |  |  |
|  | |  |  | | |  | | |  | | |  | | | |  | | |  |
| Dorminance | | 1-nma | | | 10 | | | 4.16 | | | 0.20 | | | *F* = 0.853 *p* = 0.519 *η*2 p = 0.282 | | |  | | |
|  |  | 2-cma | | | 10 | | | 4.32 | | | 0.18 | | |  |  |  |  | | |
|  |  | 3-lea | | | 10 | | | 4.33 | | | 0.18 | | |  |  |  |  | | |
|  |  | 4-nmp | | | 10 | | | 4.17 | | | 0.28 | | |  |  |  |  | | |
|  |  | 5-cmp | | | 10 | | | 4.23 | | | 0.43 | | |  |  |  |  | | |
|  |  | 6-lep | | | 10 | | | 4.31 | | | 0.24 | | |  |  |  |  | | |
|  | |  |  | | |  | | |  | | |  | | | |  | | |  |
| Intelligence | 1-nma | | | 10 | | | 4.62 | | | 0.31 | | | *F* = 22.270 *p* < 0.001 *η*2 p = 1.000 | | 1 > 2/3/4/5/6 2 > 3/4/5/6 6 < 4/5 | | |  | |
|  | 2-cma | | | 10 | | | 4.60 | | | 0.39 | | |  |  |  |  |  |  | |
|  | 3-lea | | | 10 | | | 3.72 | | | 0.26 | | |  |  |  |  |  |  | |
|  | 4-nmp | | | 10 | | | 3.89 | | | 0.38 | | |  |  |  |  |  |  | |
|  | 5-cmp | | | 10 | | | 4.00 | | | 0.32 | | |  |  |  |  |  |  | |
|  | 6-lep | | | 10 | | | 3.37 | | | 0.32 | | |  | |  |  |  |  | |
| a.        1-nma=novel metaphor targeting appearance, 2-cma=conventional metaphor targeting appearance, 3-lea=literal expression targeting appearance, 4-nmp=novel metaphor targeting possessions, 5-cmp=conventional metaphor targeting possessions, 6-lep=literal expressions targeting possessions | | | | | | | | | | | | | | | | | | |  |
| b.       *p*<0.05. | | | | | | | | | | | | | | | | | | |  |

Supplementary Table S2

| **Table S2 ▏Relationship Status (RS) and Love Attitude Scale (LAS) types. (N=114)** | | | |
| --- | --- | --- | --- |
|  | | Relationship Status | |
|  |  | In Relation (N=49) | Single  (N=65) |
| Primary type (Cramer’s *V*=0.093) | | | |
| *Storge* | Count | 28 | 24 |
|  | % within RS | 57.1% | 36.9% |
|  | % within LAS | 53.8% | 46.2% |
| *Eros* | Count | 20 | 38 |
|  | % within RS | 40.8% | 58.5% |
|  | % within LAS | 34.5% | 65.5% |
| *Ludus* | Count | 1 | 3 |
|  | % within RS | 2.0% | 4.6% |
|  | % within LAS | 25.0% | 75.0% |
|  |  |  |  |
| Secondary type (Cramer’s *V*=0.132) | | | |
| *Pragma* | Count | 30 | 38 |
|  | % within RS | 61.2% | 58.5% |
|  | % within LAS | 44.1% | 55.9% |
| *Mania* | Count | 12 | 9 |
|  | % within RS | 24.5% | 13.8% |
|  | % within LAS | 57.1% | 42.9% |
| *Agape* | Count | 7 | 18 |
|  | % within RS | 14.3% | 27.7% |
|  | % within LAS | 28.0% | 72.0% |
|  | | | |
